# *Lacticaseibacillus casei* Combats Biofilm Formation and Exhibits Antibacterial Activity Against Clinical Isolates of *Staphylococcus aureus*, *Salmonella enterica*, and *Escherichia coli*

**DOI:** 10.3390/microorganisms13122667

**Published:** 2025-11-24

**Authors:** Despoina Eugenia Kiousi, Sotiris Kyriakou, Christos Efstathiou, Stylianos Didaskalou, Maria Koffa, Aglaia Pappa, Maria Panopoulou, Mihalis I. Panayiotidis, Alex Galanis

**Affiliations:** 1 Department of Molecular Biology and Genetics, Faculty of Health Sciences, Democritus University of Thrace, 68100 Alexandroupolis, Greece; dkiousi@mbg.duth.gr (D.E.K.); cefstath@mbg.duth.gr (C.E.); sdidaska@mbg.duth.gr (S.D.); mkoffa@mbg.duth.gr (M.K.); apappa@mbg.duth.gr (A.P.); 2 Department of Cancer Genetics, Therapeutics and Ultrastructural Pathology, The Cyprus Institute of Neurology and Genetics, 2371 Nicosia, Cyprus; sotirisk@cing.ac.cy (S.K.); or mp2358@msstate.edu (M.I.P.); 3 Department of Medicine, Faculty of Health Sciences, Democritus University of Thrace, 68100 Alexandroupolis, Greece; mpanopou@med.duth.gr; 4 Department of Comparative Biomedical Sciences, School of Veterinary Medicine, Mississippi State University, Starkville, MS 39762, USA

**Keywords:** *Lacticaseibacillus casei*, antimicrobial, antibiofilm, pathogens, untargeted metabolomics, cell-free culture supernatants, probiotics

## Abstract

Biofilm-forming pathogens are a major cause of persistent infections, showing limited response to antibiotic treatment. The search for alternative strategies has therefore driven extensive research into the antimicrobial potential of beneficial microorganisms. In the present study, the antibacterial and antibiofilm activity of the commercial probiotic strain *Lacticaseibacillus casei* ATCC 393 (Lc393) was examined against clinical isolates of *Staphylococcus aureus*, *Salmonella enterica* subsp. *enterica* serovar Enteritidis and *Escherichia coli*. Lc393 reduced pathogen viability and attachment to the colon adenocarcinoma cell line HT-29, with maximal effects recorded against *S. aureus*. Confocal microscopy visualization of the lactobacilli-pathogens-host interface revealed that Lc393 binds loosely to both host cells and pathogens. The Lc393 cell-free culture supernatant (CFCS) significantly reduced planktonic growth, biofilm mass, and viability of cells in biofilm (>2 logCFU reduction, *p* < 0.05) and downregulated genes involved in the early stages of biofilm formation in *S. aureus* (i.e., *icaA, fnbpA, eno*). In silico analysis of the Lc393 genome identified two bacteriocin clusters, along with genes related to ethanol and organic acid production. Based on in silico predictions and a bacteriocin zymogram, the strain cannot produce functional antimicrobial peptides. Untargeted metabolomics based on UPLC/MS further revealed the presence of putative antimicrobial metabolites. Collectively, our findings highlight the antimicrobial potential of *Lc. casei* ATCC 393 and support its further investigation for combating clinically relevant human pathogens.

## 1. Introduction

Biofilm-associated infections are a leading cause of chronic human disease, contributing to high morbidity and mortality [1]. Antibiotics are often ineffective as biofilm matrices hinder their penetration and diffusion, while pathogens within this matrix have reduced metabolic and replication rates [2]. Treatment failures often necessitate prolonged therapy, leading to the emergence and spread of (multi-)resistant pathogens in community, hospital, as well as farming and environmental settings [3,4]. The most common antibiotic-resistant pathogen in biofilms is *S. aureus*. It is mainly associated with wound infections and implanted medical devices, frequently leading to serious complications such as sepsis and even death [5]. Similarly, certain *E. coli* pathotypes, including uropathogenic *E. coli* (UPEC), have been associated with recurrent infections, as they can effectively colonize the host epithelium, presenting low response rates to antibiotic treatments [6]. Biofilm-forming pathogens are also a major problem for the food industry [7]. A typical example is non-typhoidal *S. enterica*, which can form biofilms on food contact surfaces, increasing contamination risk and outbreaks, due to its resistance to antimicrobial agents and disinfectants [8].

The urgent need for novel strategies to combat these pathogens has driven research into microorganisms with antibacterial and antibiofilm potential, including lactobacilli. These Gram-positive bacteria have developed a range of tools to limit the colonization, adaptation, and growth of other microbes in their niche. Their antimicrobial arsenal includes organic acids, peptides, and small proteins such as bacteriocins, as well as secondary metabolites, ethanol, and reactive oxygen species (ROS) [9]. To combat biofilm formation, lactobacilli can hijack quorum sensing signals and modify the expression of key genes involved [10]. In the host niche, they can inhibit colonization and host cell invasion or indirectly modulate the infection outcomes through strain-specific effects on pathogen clearance responses [11,12].

*Lc. casei* ATCC 393 is a commercially available probiotic strain, originally isolated from Emmental cheese [13]. It has been incorporated into several dairy [14,15] and non-dairy [16] foodstuffs. Lc393 exhibits several probiotic properties: it can withstand gastrointestinal transit, transiently adhere to the murine intestinal mucosa [17], modulate resident microbiota composition [18], regulate host immune responses [19,20], exert anti-proliferative effects [21], and protect against cognitive dysfunction [22]. In addition, Lc393 has demonstrated antimicrobial activity against enteroaggregative *Escherichia coli* (EAggEC) [23], *Pseudomonas aeruginosa* [24], *Mannheimia heamolytica* [25], and invasive *Salmonella enterica* serovar *Typhimirium* [26].

In this study, the antibacterial and antibiofilm potential of Lc393 against clinical isolates of *Staphylococcus aureus*, *Salmonella enterica* serovar Enteritidis, and *Escherichia coli* was evaluated using microbiological, genomic, and metabolomic methodologies. The antibacterial activity of viable Lc393 against planktonic cells was first assessed by agar well diffusion and co-culture assays. Its capacity to limit pathogen adhesion, invasion of host cells, and pathogen-induced cell death was then determined in a cellular model of infection. The ability of Lc393 cell-free culture supernatant (CFCS) to limit planktonic growth and biofilm formation was investigated through microbiological assays and confocal microscopy, while its impact on the expression of genes related to biofilm formation was evaluated at the transcriptional level. The Lc393 genome was subsequently searched for gene clusters potentially associated with antibacterial phenotypes, and finally, untargeted metabolomics was employed to characterize the metabolite profile of Lc393 CFCS.

## 2. Materials and Methods

### 2.1. Bacterial Strains

*Lc. casei* ATCC 393 (Lc393) was acquired from ATCC (Manassas, VA, USA), *Lc. rhamnosus* GG DSM 111733 (LGG) was obtained from DSMZ (Braunschweig, Germany) and was used as a reference strain. Lactobacilli were cultivated O/N in de Man, Rogosa and Sharp (MRS) (AppliChem, Darmstadt, Germany) broth at 37 °C, under anaerobic conditions. Clinical isolates of *Staphylococcus aureus*, *Salmonella enterica* ssp. *enterica* serovar Enteritidis and *Escherichia coli* were obtained from the microbial collection of the Laboratory of Clinical Microbiology, University Hospital of Alexandroupolis, Greece [10]. Pathogens were cultured in Tryptic Soy Broth (TSB) (Condalab, Madrid, Spain) at 37 °C. Tryptone soya agar (TSA) (Condalab) and McConkey agar (VWR, Radnor, PA, USA) were used for colony enumeration of *S. aureus* or *S. enterica* and *E. coli*, respectively.

### 2.2. Eukaryotic Cell Cultures

The human colorectal adenocarcinoma cell line HT-29 was purchased from ATCC. Cells were cultured in Roswell Park Memorial Institute GlutaMAXTM (RPMI)-1640 medium, 10% fetal bovine serum (FBS), 100 μg/mL streptomycin, and 100 U/mL penicillin (all from Thermo Fisher Scientific, Waltham, MA, USA). For HT-29-bacteria co-incubations, a modified RPMI-1640 medium consisting of 10% FBS and 20 mM 4-(2-hydroxyethyl)-1-piperazineethanesulfonic acid (HEPES) (Thermo Fisher Scientific) was used. Cells were incubated in a humidified atmosphere at 37 °C, 5% CO_2_.

### 2.3. Well Diffusion Assay

The antimicrobial activity of Lc393 cultures was, initially, investigated using the well diffusion assay, as previously described [10]. MRS was used as a negative control, and kanamycin (50 μg/mL, Sigma-Aldrich, Saint Louis, MO, USA) as a positive control. Inhibition zones (cm) were measured the day after incubation at 37 °C.

### 2.4. Pathogen Growth Inhibition Assays

For the investigation of the antibacterial activity of viable Lc393, fresh O/N cultures (10^8^ CFU/mL) were co-incubated with pathogens (10^8^ CFU/mL) in TSB for 24 h at 37 °C under anaerobic conditions. The next day, the bacterial suspension was serially diluted in Ringer’s solution and plated on agar plates for colony enumeration. Pathogen cell viability is presented in logCFU/mL.

The antibacterial potential of metabolites produced by Lc393 was tested using CFCS collected by centrifugation of Lc393 cultures grown in MRS for 22 h (4000× *g*, 10 min), followed by sterile filtration and adjustment to pH 6. Planktonic pathogens (10^8^ CFU/mL) were treated with CFCS for 24 h, and MRS served as a negative control. Pathogen viability was determined spectrophotometrically at 620 nm. Results are expressed as the percentage (%) = [(Sample OD_620_ − Media blank OD_620_)/(Mean control OD_620_ − Media blank OD_620_)] × 100.

### 2.5. Aggregation Capacity

Auto- and co-aggregation capacity was evaluated using a previously published method [27], with minor modifications. To test co-aggregation capacity, 10^8^ CFU/mL of Lc393 or LGG were mixed with 10^8^ CFU/mL of each pathogen and remained at room temperature (RT) under static conditions. Lactobacilli (10^8^ CFU/mL) and pathogens (10^8^ CFU/mL) were separately tested for auto-aggregation. The upper layer of the suspension was sampled (500 μL) at 0 and 4 h. Results are expressed as: Co-aggregation (%) = [(A_1_ + A_2_)/2 − A_mix_ (A_1_ + A_2_)/2] × 100, where A_1_ and A_2_ represent the absorbance at 620 nm of pathogen and lactobacilli monocultures (auto-aggregation), respectively, and A_mix_ the absorbance of the mixed culture (co-aggregation). Auto-aggregation was calculated using the formula: 1 − (At = 0 h/A_t_ = 4 h) × 100, where A_t_ = 0 h and A_t_ = 4 h refer to absorbance at 620 nm of the monocultures at 0 and 4 h, respectively.

### 2.6. Competitive Exclusion Assay

The ability of the lactobacilli to inhibit pathogen adhesion on HT-29 cell monolayers was determined using a microbiological assay. HT-29 cells were seeded in 24-well plates (40 × 10^4^ cells per well) and incubated until the formation of a monolayer (100% confluency, ~10^6^ cells per well). Cells were then co-incubated with Lc393 or LGG (10^7^ CFU/mL, multiplicity of infection (MOI): 10) and pathogens (10^7^ CFU/mL, MOI: 10) for 4 h. Cells treated with pathogens alone served as controls. Following incubation, monolayers were washed twice with Phosphate-Buffered Saline (PBS) (Thermo Fischer Scientific) and were detached using 1× Trypsin (Thermo Fischer Scientific). The cell suspension was serially diluted in Ringer’s solution and plated onto agar plates for colony enumeration. Attached bacteria were expressed as logCFU/mL.

### 2.7. Gentamicin Protection Assay

The gentamicin protection assay was performed to investigate the capacity of Lc393 to inhibit pathogen internalization. HT-29 cells were seeded in 24-well plates at a density of 5 × 10^4^ cells per well. The next day, cells were co-incubated with lactobacilli (10^7^ CFU/mL) and pathogens (10^7^ CFU/mL) for 1 h. Cell monolayers were, then, washed twice with PBS and incubated in RPMI medium with 10% fetal bovine serum (FBS) and 100 μg/mL gentamicin (all from Thermo Fischer Scientific) for 1 h. HT-29 cells were lysed using 1% *v*/*v* Triton-X and the lysate was serially diluted in Ringer’s solution and plated for colony enumeration. Internalized bacteria are expressed as logCFU/mL. Cells treated with pathogens alone (without lactobacilli) served as a positive control.

### 2.8. Confocal Microscopy

#### 2.8.1. Sample Preparation

Confocal microscopy was utilized for the visualization of lactobacilli-pathogens-host interactions and biofilm formation. 40 × 10^5^ cells per well were seeded in 6-well plates on No. 1.5 coverslips and were incubated overnight at 37 °C with 5% CO_2_ in a humidified incubator. HT-29 cells were stained with SIR-actin (Spirochrome, Denver, CO, USA) for 6 h, according to the manufacturer’s instructions. Lactobacilli (10^7^ CFU/mL) and pathogens (10^7^ CFU/mL) were stained with 10 μΜ carboxyfluorescein succinimidyl ester (CFSE) (ThermoFisher Scientific) and 5 μΜ of CellTrace Violet cell dye (Invitrogen, Waltham, MA, USA), respectively, for 20 min at 37 °C. After staining, the suspension of lactobacilli and pathogen was co-cultured with HT-29 cells for 1 h in a humidified atmosphere at 37 °C, 5% CO_2_. For biofilm visualization, pathogens (10^8^ CFU/mL) were seeded on No. 1.5 coverslips and were treated with either MRS (pH 6) or with CFCS (pH 6, 50% *v*/*v*) for 24 h. Then, biofilms were washed twice with PBS and incubated with 10 μΜ CFSE stain (BD Biosciences, Franklin Lakes, NJ, USA) and 1 μg/mL Hoechst 33342 dye (Biotium, San Francisco, CA, USA) for 1 h. In both experimental setups, coverslips were washed three times with PBS and fixed in 4% paraformaldehyde (PFA) (AppliChem, Darmstadt, Germany) in PHEM solution [60 mM PIPES, 25 mM HEPES, 10 mM EGTA (Merck Millipore, Burlington, MA, USA, 2 mM MgCl_2_ (Applichem) pH 6.9] for 12 min at RT and were mounted in homemade mowiol 4-88 (AppliChem) medium.

#### 2.8.2. Image Acquisition

Image acquisition was performed on a customized Andor Revolution Spinning Disk Confocal system (Yokogawa CSU-X1) built around an Olympus IX81 with a 60× 1.42NA oil lens (UPlanXApo; Olympus Shinjuku, Tokyo, Japan) and an Andor Zyla 4.2 sCMOS (Andor Technology Ltd., 142 Belfast, Northern Ireland) (Bioimaging Facility, MBG-DUTH) for both biofilm and lactobacilli-pathogens-host interactions visualization. The system was controlled by Andor IQ3 software (version 3.6.4). Biofilm imaging was conducted using laser excitation lines at 405 nm and 488 nm, with corresponding camera exposure times of 0.5 s and 0.1 s, and laser intensities of 100 mW set to 100% and 65% power, respectively. For visualization of the interactions, 100 mW laser excitation lines at 405 nm, 488 nm, and 638 nm were used, with camera exposure times of 0.6 s, 0.8 s, and 1.0 s at 100% power, respectively. Hoechst 33342 and CellTrace Violet were excited with the 405 nm laser, and fluorescence was detected using a single-band HQ445/40 m (Chroma, Bellows Falls, VT, USA) emission filter. CFSE was excited with the 488 nm laser and detected using a single-band HQ525/50 m (Chroma) emission filter. SiR-actin was excited with the 638 nm laser and detected using a single-band HQ700/75 m (Chroma) emission filter. Images were acquired as z-stacks with a z-step of 0.5 μm, through the entire volume of the cells. Images of the lactobacilli-pathogens-host interface were de-convolved using the Huygens deconvolution software (version 22.10.0p6) (Scientific Volume Imaging B.V., Hilversum, The Netherlands). For each image, the maximum projection of z-stacks was generated, and the background was subtracted using a custom script in ImageJ (version 1.54f) (National Institute of Health, Bethesda, MD, USA).

### 2.9. Eukaryotic Cell Viability Assay

The protective effects of viable lactobacilli against pathogen-induced toxicity were evaluated using the sulforhodamine B (SRB) (Invitrogen, Waltham, MA, USA) assay, following a previously published protocol [11]. Briefly, HT-29 cells were seeded at a density of 7500 cells per well in a 96-well plate and the following day, cells were treated with lactobacilli (10^7^ CFU/mL) and pathogens (10^8^ CFU/mL) (co-treatments) or incubated (2 or 4 h) with lactobacilli (10^7^ CFU/mL) before the addition of pathogens (10^8^ CFU/mL) (pre-treatments). Pathogen challenge lasted for 4 or 2 h for *S. aureus* and *E. coli*, respectively. Cell survival (%) is expressed as: [(Sample OD_570_ − Blank OD_570_)/(Control mean OD_570_ − Blank OD_570_)] × 100. Cells treated with standard culture medium served as the untreated control, and cells exposed only to pathogens served as the positive control.

### 2.10. Antibiofilm Activity of Lactobacilli CFCS

For the estimation of viability of the cells within the biofilm, after treatment with lactobacilli CFCS, a microbiological assay was followed, as previously described [10]. Briefly, pathogens (10^8^ CFU/mL) were co-incubated with CFCS (pH 6, 50% *v*/*v*) in a 96-well plate for 24 h. For the estimation of viable cells, biofilms were washed twice post-treatment with PBS and mechanically disrupted. The resulting suspension was serially diluted in Ringer’s solution and spread onto agar plates. Viable biofilm-associated bacteria are presented as logCFU/mL.

### 2.11. RNA Extraction, cDNA Synthesis, and qPCR

Gene expression analysis at the transcriptome level was employed to examine changes in the expression of biofilm-associated genes following exposure to CFCS, following a previously published protocol [10]. All reactions were performed in duplicates, and each experiment included two negative controls for each primer used. Relative gene expression was calculated with the formula RQ = 2^−ΔΔCt^. The sequences of primers used in this study have been previously published [10].

### 2.12. In Silico Analysis

Bacteriocin clusters were predicted using BAGEL4 [28] and AntiSMASH v8.0 [29], BLASTp v2.16.0 [30]. The prediction of the topology and physicochemical properties of putative bacteriocins was performed using DeepTMHMM v1.0 [31] and ProtParam [32], respectively. InterPro v106.0 [33] and Signal 6.0 [34] were employed to classify the proteins into families and identify related motifs and N-terminal signals. Secondary metabolite biosynthesis clusters were annotated with AntiSMASH v8.0 and BlastKOALA v3.1 [35].

### 2.13. Metabolomics

Freeze-dried CFCS was reconstituted in ice-cold acetonitrile (800 μL) and vortexed extensively. The supernatant was isolated, and the formed precipitant was re-extracted with an additional 200 μL of acetonitrile. The combined extracts were passed through 0.22 μm filter syringe and transferred into HPLC amber vials. Samples were analyzed on an Agilent REVIDENT Quadrupole Time-Of-Flight (Q-TOF) mass spectrometer coupled with an Agilent 1290 Infinity II UPLC system via Dual AJS (ESI) electrospray ionization source (Agilent Technologies, Santa Clara, CA, USA). The Agilent 1290 Infinity II UPLC system consists of a binary pump, an online vacuum degasser, an autosampler, and a thermostat column chamber. Mass spectrometric analysis was carried out on an Agilent REVIDENT Q-TOF mass spectrometer in positive and negative electrospray ionization (ESI) mode. The autosampler temperature was set at 4 °C, and the metabolites were chromatographically separated using an Agilent InfinityLab Poroshell 120 HILIC-Z, 2.1 mm × 150 mm, 2.7 (Agilent, Santa Clara, CA, USA). The flow rate was maintained at 250 μL × min^−1^, and the sample injection volume was set at 3 μL. For ESI-positive experiments: The column temperature was maintained at 25 °C. Mobile phase A was 100% aqueous 10 mM ammonium formate acidified with 0.1% formic acid. Mobile phase B was 10% aqueous 10 mM ammonium formate: 90% acetonitrile acidified with 0.1% formic acid. Gradient was as follows: 98% B; 98% B at 3 min; 70% B at 11 min; 60% B at 12 min; 5% B at 16 min; 5% B at 18 min; 98% B at 19 min; 98% B at 20 min. The column was held at 98% B for a re-equilibration phase of 2 min at 400 μL × min^−1^ between sample injections. For ESI-negative experiments: The column temperature was maintained at 50 °C. Mobile phase A was 100% aqueous 10 mM ammonium acetate. Mobile phase B was 15% aqueous 10 mM ammonium acetate: 85% acetonitrile. Gradient was as follows: 96% B; 96% B at 2 min; 88% B at 5.5 min; 88% B at 8.5 min; 86% B at 9 min; 86% B at 14 min; 82% B at 17 min; 65% B at 23 min; 65% B at 24 min; 96% B at 24.5 min; 96% B at 26 min. The column was held at 96% B for a re-equilibration phase of 2 min at 400 μL × min^−1^ between sample injections.

The resolving power of the Q-TOF analyzer was set above 10,000 (FWHM, with full width at half maximum), and spectra were acquired within a mass range of *m*/*z* 60–1000 (ESI-positive) and *m*/*z* 60–1600 (ESI-negative). Nitrogen gas at 225 °C, 6 L/min, 40 psi (ESI-positive) and 13 L/min, 35 psi (ESI-negative) was used for nebulizing and drying in the ionization source. The sheath gas was maintained at 225 °C with a flow rate of 10 L/min (ESI-positive) and at 350 °C with a flow rate of 12 L/min (ESI-negative). The fragmentor was set at 125 V (ESI-positive) and at 110 V (ESI-negative), and the skimmer at 65 V (ESI-positive) and at 45 V (ESI-negative). The capillary, nozzle and octapole 1 RF voltage was set at 3000 V, 0 V, and 450 V, respectively (ESI-positive) and at 3500 V, 0 V, 750 V, respectively (ESI-negative). Data were acquired under continuous reference mass correction using purine (*m*/*z* 121.0509 for ESI-positive and *m*/*z* 119.0363 for ESI-negative) and HP-0921 (*m*/*z* 922.0890 for ESI-positive and *m*/*z* 980.0163 for ESI-negative).

Analysis was conducted using Agilent MassHunter Explorer (v1.0). Formula targets were based on both positive (H^+^) and negative (H^−^) ion species, with charge states limited to a range of 1–2. Mass and retention time (RT) tolerances were set to ±10 ppm + 2 mDa and ±0.00% + 50.5 min, respectively. Features with an absolute intensity below 1000 or saturation greater than 20% were excluded from the analysis. Detected metabolite features were required to meet these conditions across all sample groups. Compounds identified in the extraction blanks were omitted. Metabolite abundances were calculated based on ion peak areas, and the resulting values were exported as .CSV files for downstream analysis and visualization. Only metabolites showing a fold change of ≥2 and a *p* < 0.05 were considered statistically significant. Tentative metabolite identifications were assigned using the Agilent MassHunter METLIN Metabolomics Database (curated in Agilent PCDL Manager, version B.08.00). Compounds were first annotated via library/database search; when no matches were found, molecular formula generation was used. LC/MS tolerances for precursor ion *m*/*z* were set at ±10 ppm + 2 mDa. Identification scoring parameters (database search and molecular formula generation scores) were evenly weighted at 40. Search results were restricted to the top 5 hits per compound, with scores above 70 deemed reliable.

### 2.14. Statistical Analysis

Student’s *t*-test was performed for the statistical analysis of experimental data using GraphPad PRISM v9.0 (GraphPad Software Inc., San Diego, CA, USA). All experiments were performed in triplicate. Results are represented as mean ± standard deviation. A *p* < 0.05 was considered statistically significant.

## 3. Results

### 3.1. Viable Lc393 Exerts Antibacterial Activity

**Figure 1:**
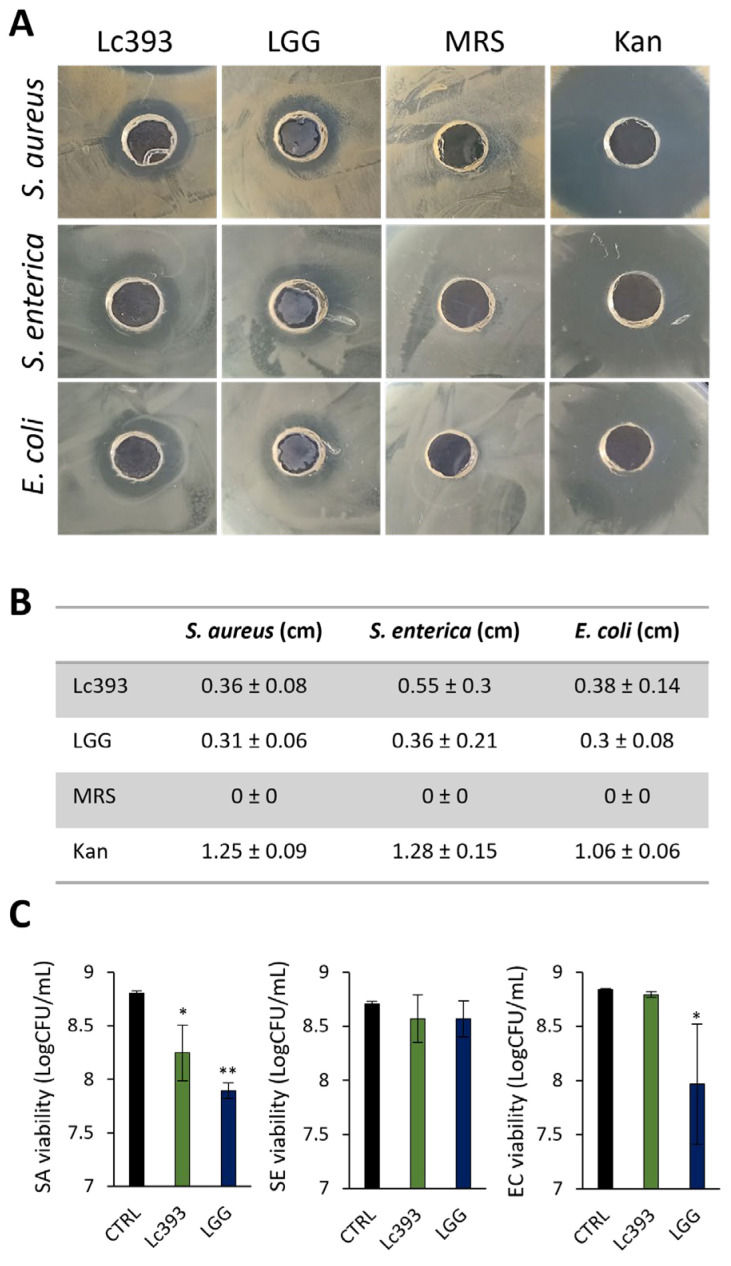
Evaluation of the antibacterial activity of viable lactobacilli against *S. aureus* (SA), *S. enterica* (SE), and *E. coli* (EC). (**A**) Representative images of inhibition zones produced by fresh O/N cultures of *Lc. casei* ATCC 393 (Lc393), *Lc. rhamnosus* GG (LGG), MRS (negative control), and kanamycin (Kan, positive control) against SA, SE, and EC, using the agar well diffusion assay. (**B**) Zone of inhibition in cm. Results are expressed as mean ± standard deviation of three independent experiments. (**C**) Antibacterial activity of viable lactobacilli against planktonic pathogens in suspension. Viability was measured after 24 h of lactobacilli-pathogen co-incubation and is expressed as logCFU/mL. Results are expressed as mean ± standard deviation of three independent experiments. * *p* < 0.05, ** *p* < 0.01, compared to untreated samples (CTRL).

Using the agar well diffusion assay, it was shown that viable Lc393 inhibited the growth of *S. aureus, S. enterica,* and *E. coli* (Figure 1A,B). In suspension, Lc393 significantly limited *S. aureus* viability (*p* < 0.05), having no effect against *S. enterica* or *E. coli*, while LGG reduced the growth of both *S. aureus* and *E. coli* (Figure 1C).

### 3.2. Investigation of Lc393–Pathogens–Host Interactions

**Figure 2:**
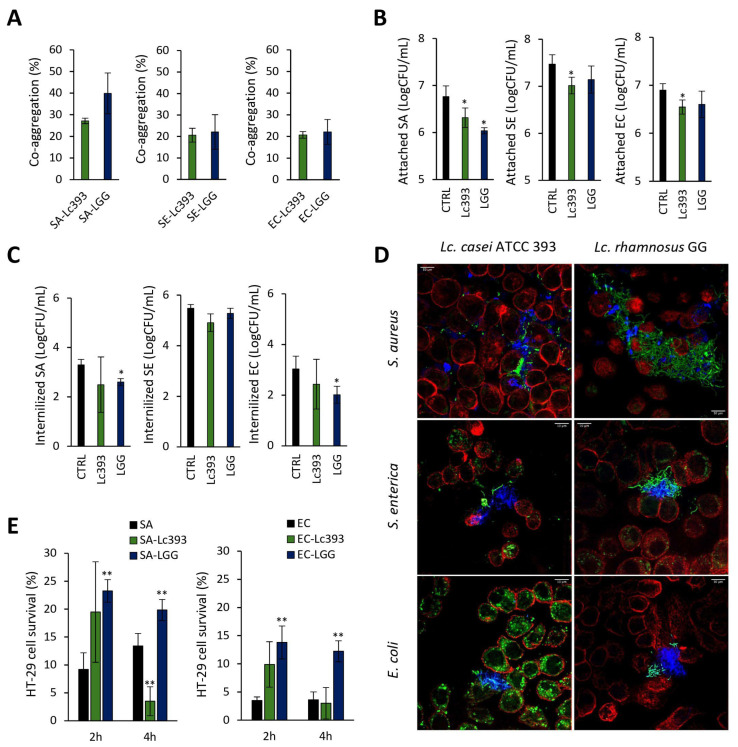
Investigation of lactobacilli-pathogens-host interactions. (**A**) Evaluation of the co-aggregation capacity of *Lc. casei* ATCC 393 (Lc393) and *Lc. rhamnosus* GG (LGG) with *S. aureus* (SA), *S. enterica* (SE), and *E. coli* (EC) after 4 h co-incubation in suspension, under static conditions. (**B**) Effect of lactobacilli on pathogen attachment to HT-29 cells. (**C**) Effect of lactobacilli on pathogen internalization into HT-29 cells. Results are expressed as mean ± standard deviation of three independent experiments. * *p* < 0.05. (**D**) Confocal microscopy visualization of lactobacilli–pathogens–HT-29 interactions. HT-29 cells are stained with SIR-actin (red), lactobacilli with CFCS (green), and pathogens with CellTrace Violet (blue). Scale bar 10 μm. (**E**) Assessment of the protective role of lactobacilli treatments against pathogen-induced cell death. HT-29 cells were pre-incubated with lactobacilli for 2 or 4 h before the addition of SA or EC. Cell viability was determined after incubation with the pathogens for 4 h (SA) or 2 h (EC) using the SRB assay. Data are expressed as mean ± standard deviation of three independent experiments. * *p* < 0.05 and ** *p* < 0.005 compared to pathogen-only control cells (SA or EC).

Lc393 co-aggregated with the three pathogens at similar rates (20–28%) (Figure 2A). In a cellular model of infection, Lc393 significantly excluded the pathogens from HT-29 cell monolayers (~0.5 log CFU/mL, *p* < 0.05) (Figure 2B) but did not affect pathogen internalization (Figure 2C). LGG significantly limited both *S. aureus* attachment and the internalization of *S. aureus* and *E. coli* (*p* < 0.05). These interactions were visualized by confocal microscopy. As shown in Figure 2D, LGG formed net-like structures engulfing the pathogens, while Lc393 exhibited lower co-aggregation capacity and adherence to the host cell surface. At this timepoint, no significant decrease in pathogen viability was recorded.

We next assessed the capacity of Lc393 to protect HT-29 cells from pathogen-induced cell death under two experimental conditions: (i) co-incubation with the lactobacilli and pathogens for 4 h (*S. aureus*-treated cells) or 2 h (*E. coli*-treated cells), and (ii) pre-incubation with lactobacilli for 2 or 4 h before the addition of pathogens. As presented in Figure 2E and Figure S1, Lc393 did not protect host cells from cell death in either condition. It should be noted that cells that were pre-incubated with Lc393 for 4 h before the addition of *S. aureus* exhibited increased loss of cell viability compared to pathogen-only treated cells (Figure 2E). As expected, LGG significantly limited *S. aureus* and *E. coli*-induced cell death, in agreement with previous findings from our group [11].

### 3.3. Lc393 CFCS Inhibits Planktonic Pathogen Viability and Biofilm Formation Capacity

**Figure 3:**
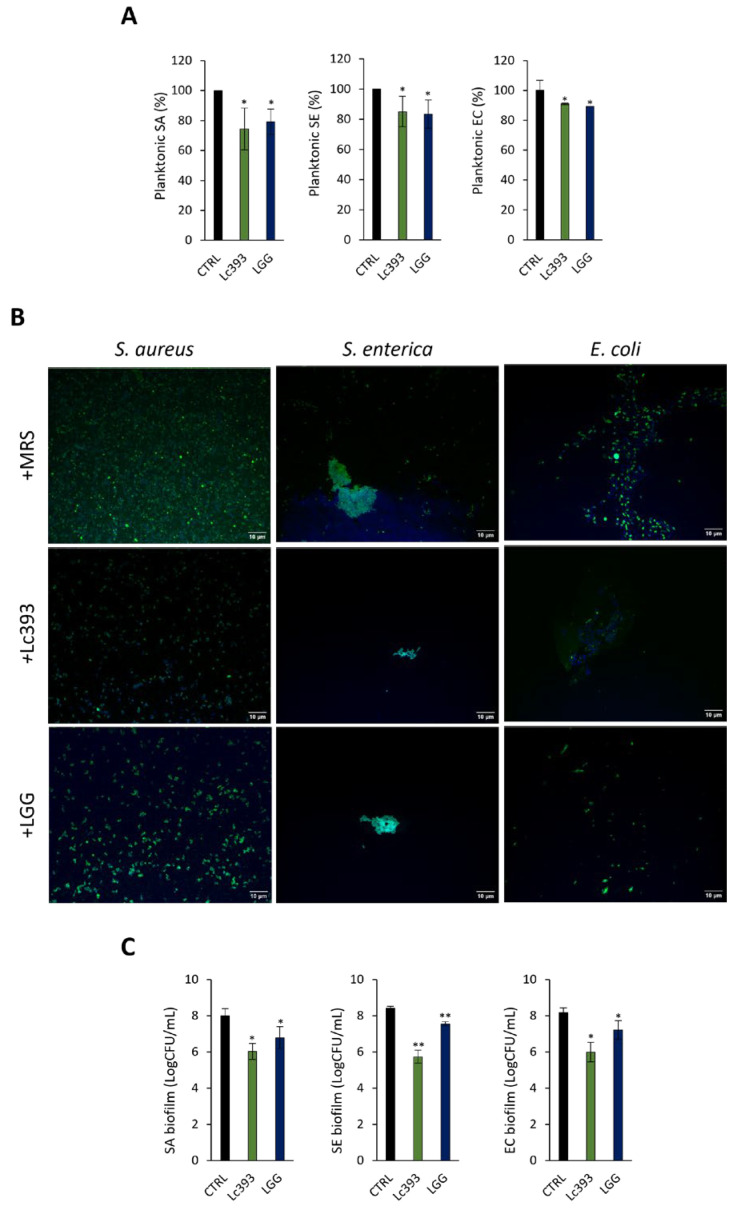
Antibacterial and antibiofilm activity of lactobacilli-derived CFCS. (**A**) Effect of lactobacilli CFCS on *S. aureus* (SA), *S. enterica* (SE) and *E. coli* (EC) viability after 24 h incubation at 37 °C. Viability was determined spectrophotometrically at 620 nm. Data are presented as the mean ± standard deviation of three independent experiments. * *p* < 0.05 compared to untreated pathogens (CTRL). (**B**) Representative confocal images of pathogen biofilms after 24 h incubation in 50% *v*/*v* MRS (control) or CFCS (50% *v*/*v*, pH 6) derived from *Lc. casei* ATCC 393 (Lc393) or *Lc. rhamnosus* GG (LGG). Pathogens were stained with CFSE (green) and DNA with Hoechst 33342 (blue). Scale bar, 10 μm. (**C**) Quantitative results of the antibiofilm activity of CFCS against SA, SE and EC. Biofilm viability is expressed as logCFU/mL. Results are expressed as mean ± standard deviation of three independent experiments. * *p* < 0.05, ** *p* < 0.01 compared to CTRL.

**Figure 4:**
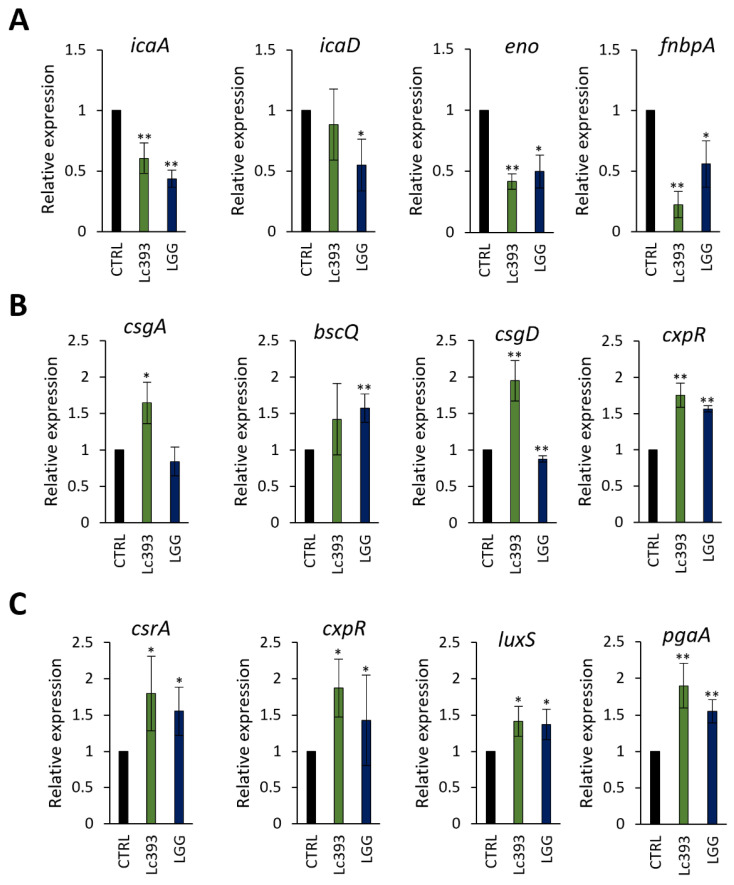
Expression levels of biofilm-associated genes in pathogens following CFCS treatment. Relative expression levels of biofilm-related genes in (**A**) *S. aureus*, (**B**) *S. enterica* and (**C**) *E. coli* after treatment with *Lc. casei* ATCC 393 (Lc393) or *Lc. rhamnosus* GG (LGG) CFCS (pH 6, 50% *v*/*v*) for 24 h. Control sample was treated with uninoculated MRS medium and TSB (1:1). Data are presented as mean ± standard deviation of three independent experiments. * *p* < 0.05; ** *p* < 0.01, compared to untreated pathogens (CTRL).

CFCS derived from Lc393 cultures significantly limited the viability of all three pathogens in suspension (Figure 3A) in a dose-dependent manner (Table S1). Accordingly, decrease in both biofilm mass (Figure 3B) and viability of biofilm-associated pathogens (>2 logCFU; *p* < 0.05) (Figure 3C) was observed. At the transcriptomic level, the expression levels of *S. aureus* adhesin genes (e.g., *eno* and *fnbpA*), as well as the master biofilm formation regulator *icaA* (Figure 4A) were downregulated, while adhesins (i.e., *csgA*, *pgaA*) and biofilm-related transcriptional regulators (i.e., *csgD*, *cxpR*, *csrA*) were upregulated in *S. enterica* (Figure 4B) and *E. coli* (Figure 4C).

### 3.4. In Silico Analysis of Genes and Genetic Clusters Coding for Bacteriocins and Antimicrobial Metabolites

**Figure 5:**
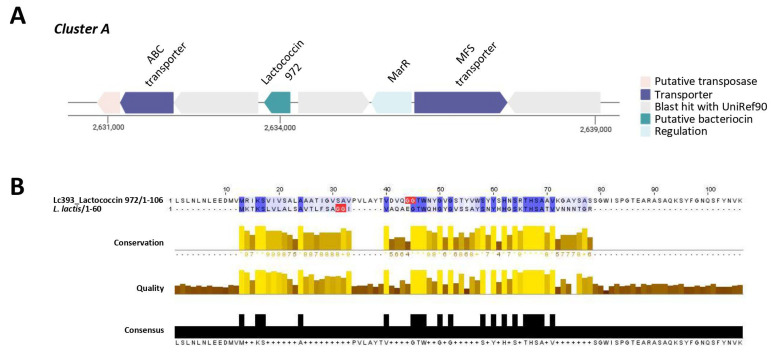
Identification of putative bacteriocin cluster A in the Lc393 genome with BAGEL4 and AntiSMASH. (**A**) Cluster A contains a gene for the core peptide lactococcin 972, an ABC-transporter, an MFS transporter, and MarR transcriptional regulator. (**B**) The core peptide (lactococcin 972) presents low sequence identity and modest structural conservation compared with the functional peptide derived from *L. lactis* IPLA 972. Sequence alignments were visualized using Jalview v2.11.4.1. Percentage identity is displayed with blue; dark blue denotes (%) > 80%. Alignment conservation is visualized as a histogram giving the score for each column. * Indicates conserved columns (score of 11); + indicates columns with mutations where all properties are conserved (score of 10). Quality is plotted on a scale from 0 to 1. The consensus sequence is shown in black. The double-glycine (GG) leader sequence is highlighted in red.

**Figure 6:**
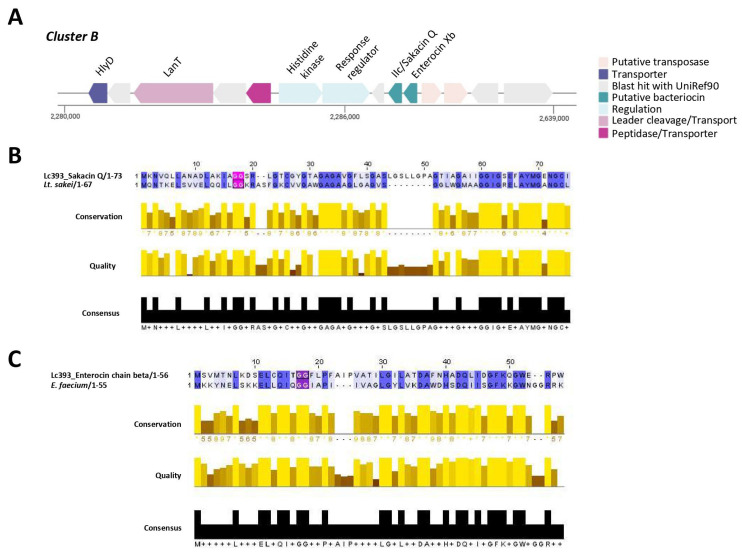
Identification of putative bacteriocin cluster Β in the Lc393 genome with BAGEL4 and AntiSMASH. (**A**) Cluster B contains two putative bacteriocins: a Blp family class II bacteriocin/sakacin Q and an enterocin X chain beta. Genes for transporters (HlyD, LanT), a protease for bacteriocin maturation (cleavage at the GG-site), and regulatory proteins (two-component system) are also present in the cluster. (**B**) Bacteriocin class II/Sakacin Q presents modest sequence and structural conservation with sakacin Q produced by *Lt. sakei* and (**C**) Enterocin chain B with the functional peptide derived from *E. faecium*. Sequence alignments were visualized using Jalview v2.11.4.1. Percentage identity is displayed with blue; dark blue denotes (%) > 80%. Alignment conservation is visualized as a histogram giving the score for each column. * Indicates conserved columns (score of 11); + indicates columns with mutations where all properties are conserved (score of 10). Quality is plotted on a scale from 0 to 1. The consensus sequence is shown in black. The double-glycine (GG) leader sequence is highlighted in pink/purple.

The genome of Lc393 was reannotated to pinpoint loci involved in the production of bacteriocins and antimicrobial metabolites. Two putative bacteriocin clusters (RiPP-like) were identified using BAGEL4 and antiSMASH (Figure 5 and Figure 6). Cluster A (location in the genome 1,903,845–1,914,129 nt, total: 10.285 nt) contains a gene encoding the core peptide lactococcin 972, an ABC-transporter, an MFS transporter, and MarR transcriptional regulator (Figure 5A). This cluster is identical to that contained in the genome of *Lc. casei* MGB0470, while the gene encoding lactococcin 972 is also conserved in other lactobacilli, including *Lactobacillus intestinalis*, and the distantly related *S. equorum* (Figure S2). The lactococcin peptide encoded by the Lc393 presents characteristics of a functional bacteriocin, including a GG N-terminal sequence, signals for extracellular localization, and a theoretical pI of 8.97 (Table 1). Although it presents 100% genome similarity with putative bacteriocins produced by *Lacticaseibacillus* spp., it presents a low percentage identity (<30%) with the functional peptide derived from *Lactococcus lactis* IPLA 972 (Figure 5B).

Two core peptides were identified in Cluster B (location in the genome 2,277,178–2,294,335 nt., total: 17,158 nt): a Blp family class II bacteriocin/sakacin Q and enterocin X chain beta (Figure 6A). Cluster B is also conserved in *Lc. casei* MGB0470 (Figure S2). The mature bacteriocin II peptide presents ~70% sequence identity and high structural conservation with sakacin Q produced by *Lactobacillus curvatus* ACU-1 (Figure 6B). Moreover, genes for bacteriocin transport (HlyD, LanT) and genes coding for two-component signal transduction systems were annotated upstream of the putative bacteriocin. The gene for enterocin Xb contained in cluster B presents a lower % identity (58.33%) with the characterized peptide derived from *Enterococcus faecium* KU-B5 (Figure 6C). Neither putative sakacin Q nor enterocin Xb present physicochemical characteristics resembling bacteriocins, while they possess intracellular and membrane-associated domains that are not commonly encountered in these peptides (Table 1). Using bacteriocin zymograms we evaluated the capacity of excreted peptides to limit pathogen viability; however, no inhibitory activity was observed (Figure S3).

The genome sequence of Lc393 was searched for genes involved in quorum quenching. Sequences for N-acyl homoserine (AHL) lactonase, AHL acylases, and AHL oxidoreductases derived from lactobacilli were identified from Uniprot. Two sequences (A0A7Z2PEZ0 and A0A0M3QBV3) of AHL lactonases encoded by *Lactiplantibacillus plantarum* were queried against the genome of the strain; however, no significant nucleotide identity was recorded (Table S2). Finally, genes coding for enzymes involved in the production of lactic acid (L-/D-lactate dehydrogenase), ethanol (decarboxylase, alpha-acetolactate decarboxylase, diphosphomevalonate decarboxylase) and hydrogen peroxide (NADH oxidase, multicopper oxidase), were identified in the genome of Lc393 (Table S3).

### 3.5. Untargeted Metabolomics

**Figure 7:**
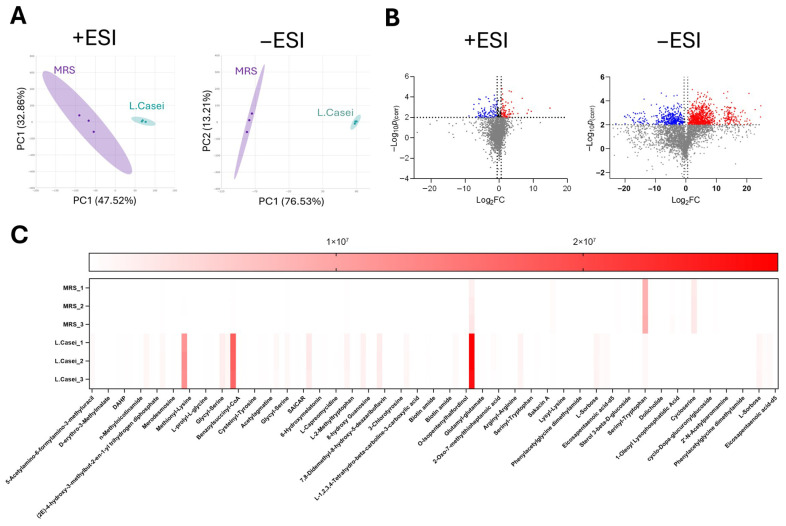
Untargeted metabolomics of Lc393 CFCS. (**A**) PLS-DA plots of the metabolite features detected in Lc393 CFCS relative to MRS, obtained under both ESI+ and ESI- mode. (**B**) Volcano plots generated to identify statistically significant features between MRS Lc393 CFCS with a fold change of >2, *p* < 0.05. The features either originate from the ESI+ or ESI− mode. Blue dots demonstrate downregulated features, red dots show upregulated metabolites relative to MRS control, and gray dots represent features that failed to pass the statistical threshold. (**C**) Heat map illustrating the peak intensities of the statistically significant features in Lc393 CFCS compared to the respective MRS control.

For the investigation of the contents of the Lc393 exometabolome (*n* = 3 batch cultivations), a Hydrophilic Liquid Chromatography (HILIC) methodology was employed, according to which the metabolites were ionized under both positive and negative ESI ionization modes. In total, 826 negatively and 345 positively (Figures S4 and S5) ionized features were detected using the Agilent MassHunter Explorer software (v2.0) operating with the ChemVista Spectrum Library. To ensure annotation reliability, only features with an absolute intensity above 1000 and annotation confidence scores above 70 (derived from database/library search or molecular formula generation) were retained for further analysis (Figure 7A). These features were subsequently subjected to PLS-DA to identify those with the highest variable importance scores. To refine the dataset, volcano plot analysis was applied to compare groups based on statistical significance (fold change ≥ 2, *p* < 0.05). In total, 96 statistically significant features were isolated and plotted in heatmaps (Figure 7C) to visualize their distribution. Among the identified metabolites, several candidates with antimicrobial potential were detected, including allantoic acid (a precursor of allantoin), isochorismate, clavamycin D, tetracenomycin A2, and actinonin (Table 2). These compounds may contribute to the antimicrobial activity of the CFCS recorded against *S. aureus*, *S. enterica,* and *E. coli*.

## 4. Discussion

The identification and characterization of microorganisms with significant antibacterial and antibiofilm activity is a top research priority. In this context, it has recently been demonstrated that *Paenibacillus* sp., a microbe found in soil microbial communities, produces a novel lasso peptide with broad-spectrum effects against a range of bacterial pathogens both in vitro and in a mouse model of *Acinetobacter baumannii* infection [36]. Furthermore, LGG, one of the most studied probiotic bacteria strains, can limit pathogen viability by matrix acidification [37], secretion of small peptides [38] or cell-surface exposed proteins [39], and by reducing pathogen adherence and invasion both in vitro [10,40,41] and in vivo [42,43]. In this study, employing LGG as a reference strain, the antibacterial and antibiofilm potential of *Lc. casei* ATCC 393 against common human pathogens was evaluated using various experimental models and omic approaches.

First, we investigated the interactions of lactobacilli with pathogenic microorganisms in vitro. Viable Lc393 significantly inhibited the growth of *S. aureus* in suspension, without having any effect on *S. enterica* and *E. coli*. In this context, we have previously demonstrated that potential probiotic lactobacilli *Lp. plantarum* L125, *Lp. pentosus* L33 and *Lc. paracasei* SP5 were more effective against Gram-positive (*S. aureus*) than against Gram-negative bacteria (*S. enterica* and *E. coli*) [10]. These results suggest that *S. enterica* and *E. coli* could affect the viability and adaptation of lactobacilli in co-cultures. For example, *E. coli* can produce a variety of antimicrobial metabolites, mainly microcins [44], while *S. enterica* can effectively compete for nutrient sources [45]. Here, *E. coli* treatments marginally decreased Lc393 viability in suspension (Figure S6). We then characterized the interactions between Lc393 and pathogens in a cellular infection model. Lc393 reduced the adhesion of all three pathogens, without affecting their internalization capacity. By contrast, the reference strain LGG effectively limited internalized counts of *S. aureus* and *E. coli*, in agreement with previous studies [10,41,46]. Microscopic visualization using confocal imaging provided critical mechanistic insights: unlike LGG, which forms dense aggregates enveloping pathogens, Lc393 maintained loose associations with host cells and microbes, consistent with a primarily secretory mode of action. Despite the limited interactions between Lc393 and pathogens at the host niche, we assessed whether Lc393 could protect host cells from pathogen-induced toxicity. The clinical isolates of *S. aureus* and *E. coli* used in this study have previously been shown to induce time-dependent HT-29 cell death after 4 and 2 h of co-incubation, respectively [11]. *S. enterica* causes cell death only after prolonged incubation (>8 h), at timepoints when lactobacilli themselves reduced cell survival. For this reason, *S. enterica* was excluded from the analysis. No protective effect was observed in cells treated with viable Lc393 under either co-incubation or pre-incubation conditions, consistent with previous studies on Lc393 and enterohemorrhagic *E. coli* (EHEC) [47], *Listeria monocytogenes* [48], and enteroaggregative *E. coli* (EggAEC) [23]. These findings collectively suggest that although Lc393 can inhibit *S. aureus* growth in suspension, it lacks the capacity to efficiently interfere with the host–pathogen interface or protect HT-29 cells from pathogen-induced cytotoxicity. The use of other cell models, including keratinocytes for *S. aureus* and bladder cell lines for *E. coli,* could provide additional insight into the ability of Lc393 to block infection in vitro and ex vivo.

Lactobacilli exert their antimicrobial activity through the production of metabolites. Most prominently, during fermentation lactobacilli secrete organic acids at high amounts outcompeting the growth of other microbes in their matrix [9]. In this context, genes involved in the production of small antimicrobial metabolites, including a range of organic acids, were annotated in the genome of Lc393. Notably, these metabolites were previously identified in late-stage fermentation Lc393 CFCS by NMR spectroscopy. Here, native CFCS (pH 4) derived from Lc393 significantly reduced pathogen viability in a dose-dependent manner (Table S4), and abolished biofilm formation. To exclude the effects of organic acids and matrix acidification, we focused on the effects of neutralized Lc393 CFCS against the pathogens and found that it suppressed both planktonic pathogen growth and biofilm formation. No pathogen-specific pattern was observed, as CFCS reduced planktonic cell and biofilm viability of all three pathogens at similar rates (~2 log units, *p* < 0.05). Previous studies have shown that lactobacilli-derived CFCS is mainly effective against *S. aureus* [49,50,51], *S. mutans* [52,53], *S. epidermidis* [54] and *L. monocytogenes* [51]. There is limited data on their effects against biofilms of Gram-negative pathogens, except for *Pseudomonas aeruginosa* [50,55]. We are currently investigating the translational potential of our findings in the context of medical device-related infections, with a particular emphasis on arthroplasty implants.

Biofilm formation is mediated by cell–cell communication and changes in the gene expression profile of planktonic pathogens, which collectively promote aggregation, adherence, and metabolic reprogramming [56]. To explore whether Lc393 metabolites interfere with this process, we evaluated the expression levels of biofilm-associated genes in *S. aureus, S. enterica,* and *E. coli* after Lc393 CFCS exposure. In *S. aureus,* CFCS significantly downregulated genes involved in intercellular adherence and attachment on surfaces, including *icaA*, (responsible for poly-beta-1,6-N-acetyl-D-glucosamine synthesis [57], *enolase* (a moonlighting adhesin), and a gene encoding a fibronectin-binding protein. Similarly, the CFCS of *L. kefiranofaciens* DD2 was previously shown to inhibit biofilm formation by oral pathogens, not only due to its antibacterial activity, but also by modulating the expression of key genes involved in biofilm formation, including genes that promote carbohydrate metabolism and adhesion, as well as genes involved in their transcriptional regulation [58]. It should be noted that treatment of *S. enterica* and *E. coli* with Lc393 CFCS upregulated several biofilm-related genes. The differential gene expression patterns observed in treated biofilm suggest pathogen-specific modes of action that merit further investigation.

Finally, we performed in silico analysis and untargeted metabolomics to gain insight into the exometabolome of Lc393. Genome analysis showed that Lc393 contains two putative bacteriocin clusters with the predicted core peptides showing limited sequence and structural conservation with previously characterized bacteriocins. This is consistent with a previous genomic study on the *Lc. casei* species [13], while, at present, there are no reports of functional bacteriocin secretion in vitro and/or in situ. In this context, we did not detect any inhibitory peptides or proteins against the tested pathogens with a modified bacteriocin zymogram. These findings suggest that the activity of Lc393 is likely metabolite-driven. Using untargeted metabolomics, we detected putative other antimicrobial metabolites that may contribute to the bactericidal phenotype described in this study. Specifically, allantoic acid is a precursor of allantoin with direct antimicrobial activity that has been investigated mostly in the context of wound healing [59]. Tetracenomycin A2 is a polyketide antibacterial compound that acts mainly by binding to bacterial ribosomes [60], whereas clavamycin D exhibits potent anticandidal activity by binding to and disrupting fungal cell membrane [61]. Actinonin is a pseudo-peptide with broad-spectrum antimicrobial activity [62], acting as an inhibitor of peptide deformylase, a prokaryotic enzyme involved in the initiation of translation [63]. Finally, isochorismate is a chelating agent for Fe^2+^ that may be involved in competition between microbial strains. It should be noted that, while this study used a high-resolution, untargeted LC-MS metabolomic approach, there are inherent methodological limitations that may have affected metabolite coverage. Specifically, highly polar, low-molecular-weight compounds, such as organic acids, elute near the void volume in HILIC columns and are poorly ionized under ESI conditions. In addition, volatile compounds, small aldehydes, and low-abundance amines may also be insufficiently detected. Furthermore, available databases may not adequately cover metabolites produced by lactobacilli, revealing a lack of data on their primary and secondary metabolism. As this was an untargeted discovery-level metabolomic analysis, the metabolite annotations were not confirmed by targeted MS/MS fragmentation. Consequently, they are considered putative and classified under MSI Level 2–3 confidence [64,65,66]. The integration of complementary platforms, such as gas chromatography-mass spectrometry (GC-MS), reverse-phase LC-MS, and targeted quantification strategies, will provide a more comprehensive profile of the Lc393 exometabolome and of its active antimicrobial components. Additionally, the loose co-aggregation pattern observed by confocal microscopy warrants exploration with super-resolution methods, such as SIM or STORM, to resolve sub-micron proximity of probiotic and pathogen surfaces. Quantitative live-cell imaging using FRAP could measure real-time metabolite dynamics and validate mechanisms of biofilm penetration and disruption. Integrating multi-omics with high-resolution imaging will accelerate the translation of probiotic exometabolomes into novel antimicrobial interventions.

## 5. Conclusions

Our study characterized the effects of viable *Lc. casei* ATCC 393 and its metabolites on the viability, biofilm formation, and infectivity of clinical isolates of *S. aureus*, *S. enterica,* and *E. coli*. Lc393 reduced pathogen viability and adhesion to the HT-29 colon epithelial cells, while not affecting pathogen internalization or pathogen-induced cytotoxicity. Significant antibacterial activity was recorded for Lc393 CFCS against all three planktonic and biofilm-associated pathogens. For *S. aureus* in particular, a modulation of the expression of genes involved in the early stages of biofilm formation was also recorded at the transcriptional level. In silico analysis and untargeted metabolomics revealed that Lc393 harbors genes for bacteriocin synthesis with low percentage identity to functional homologues but produces a range of small antimicrobial metabolites that likely contribute to the observed phenotypes. In conclusion, our results demonstrate the antimicrobial potential of Lc393 and warrant further investigation into its mechanisms of action against biofilm-forming bacteria.

## Figures and Tables

**Table 1:** Characteristics of putative bacteriocin peptides annotated in the genome of Lc393.

Putative Bacteriocin	Length (aa)	Theoretical pI	Molecular Weight (kDa)	Protein Family Membership	Signal Seq	Topology
Lactococcin 972	105	8.97	11.2	Bacteriocin, lactococcin 972 (IPR006540)	Sec/SPI	Outside
Bacteriocin II/Sakacin Q	73	5.93	6.9	None predicted	Other	Outside (but with transmembrane regions)
Enterocin Xb	56	4.52	6.2	None predicted	Other	Outside (but with transmembrane regions)

**Table 2:** Upregulated putative antimicrobial metabolites detected in Lc393 CFCS by untargeted metabolomics.

Metabolite	Theoretical *m*/*z*	Actual *m*/*z*	Retention Time (min)	Log_2_Fold of Change	*p*_corr_
Allantoic acid	176.0546	176.0514	15.363	7.137223	1.86781 × 10^5^
Tetracenomycin A2	422.4056	422.4053	7.154	3.459779	0.000147944
Isochorismate	226.0478	226.0479	7.209	5.389228	0.000267169
Clavamycin D	313.1430	315.1456	3.417	14.94609	0.003409505
Actinonin	385.2539	385.2537	21.841	11.01422	0.004445146

## Data Availability

The original contributions presented in this study are included in the article/Supplementary Material. Further inquiries can be directed to the corresponding author.

## Supplementary Materials

The following supporting information can be downloaded at https://www.mdpi.com/article/10.3390/microorganisms13122667/s1: Figure S1: Assessment of the protective role of lactobacilli treatments against pathogen-induced cell death. Cells were co-incubated with lactobacilli and pathogens for 4 or 2 h for *S. aureus* (SA) or *E. coli* (EC), respectively. Viability was determined after incubation with the SRB assay. The data presented is the mean ± standard deviation of three independent experiments; Figure S2: Homology of putative bacteriocin clusters contained in the genome of Lc393 with that contained in closely- or more distantly related bacteria. The annotation of these clusters was achieved with AntiSMASH; Figure S3: Modified SDS-PAGE assay (zymogram) for the investigation of the contribution of proteins in the antimicrobial effects recorded. Soft-agar diffusion assay for peptides contained in the CFCS of the three lactobacilli against *S. aureus*, *S. enterica*, and *E. coli* cells; Figure S4: HPLC chromatograms showing the separation of MRS or Lc393 CFCS metabolites (*n* = 3) under positive ESI mode on a HILIC column. The y axis denotes the ion abundance, whereas the x axis shows the elution time (retention time) in minutes; Figure S5: HPLC chromatograms showing the separation of MRS or Lc393 CFCS metabolites (*n* = 3) under negative ESI mode on a HILIC column. The y-axis denotes the ion abundance, whereas the x-axis shows the elution time (retention time) in minutes; Figure S6: Effect of pathogen-co-incubation to Lc393 viability. Lc393 and pathogens (*S. aureus,* SA; *S. enterica,* SE; *E. coli,* EC) were co-incubated in TSB. Viability was measured after 24 h and is expressed as logCFU/mL. Results are expressed as mean ± standard deviation of three independent experiments. * *p* < 0.05, compared to untreated samples (Lc393). Table S1: Dose-dependent effect of lactobacilli CFCS on planktonic pathogen viability; Table S2: Sequence identity of genes coded by Lc393 with N-acyl homoserine (AHL) lactonase sequences derived from Uniprot (A0A7Z2PEZ0 and A0A0M3QBV3). Calculation of percentage identity was performed using Blastp+; Table S3: Functional annotation of genes involved in the production of small antimicrobial peptides; Table S4: Dose-dependent effect of lactobacilli CFCS (pH 4) on planktonic pathogen viability.
