# Supplementary material for: Lacticaseibacillus casei Combats Biofilm Formation and Exhibits Antibacterial Activity Against Clinical Isolates of Staphylococcus aureus, Salmonella enterica, and Escherichia coli
Source: Microorganisms. 2025 Nov 24;13(12):2667. doi: 10.3390/microorganisms13122667 (PMC12735342; doi:10.3390/microorganisms13122667)
Supplement: Supplementary file 1 [file microorganisms-13-02667-s001.zip › Table S1.pdf]

Table S1. Dose-dependent effect of lactobacilli CFCS on planktonic pathogen viability.

	**Survival (%)**		
**CFCS (pH 6)**	***S. aureus***	***S. enterica***	***E. coli***
***Lc. rhamnosus* LGG**			
Undiluted	76.72 ± 7.32	89.8 ± 2.18	84.64 ± 11.5
1:2	80.90 ± 1.31	101.58 ± 5.56	76.08 ± 11.66
1:4	89.16 ± 2.84	102.57 ± 9.46	75.59 ± 13.6
1:6	90.71 ± 5.76	140 ± 9.87	99.35 ± 8.63
***Lc. casei* ATCC 393**			
Undiluted	74.28 ± 7.3	92.52 ± 2.78	79.63 ± 11.66
1:2	86.60 ± 3.23	96.74 ± 0.96	80.76 ± 10.53
1:4	85.71 ± 7.33	98.32 ± 1.27	83.18 ± 9.41
1:6	87.84 ± 13.59	116.22 ± 7.13	98.04 ± 5.87
