# Supplementary material for: Lacticaseibacillus casei Combats Biofilm Formation and Exhibits Antibacterial Activity Against Clinical Isolates of Staphylococcus aureus, Salmonella enterica, and Escherichia coli
Source: Microorganisms. 2025 Nov 24;13(12):2667. doi: 10.3390/microorganisms13122667 (PMC12735342; doi:10.3390/microorganisms13122667)
Supplement: Supplementary file 1 [file microorganisms-13-02667-s001.zip › Table S4.pdf]

Table S4. Dose-dependent effect of lactobacilli CFCS (pH 4) on planktonic pathogen viability.

	**Survival (%)**		
**CFCS (pH 4)**	***S. aureus***	***S. enterica***	***E. coli***
***Lc. rhamnosus* LGG**			
Undiluted	24.43 ± 3.4	51.05 ± 6.76	51.83 ± 6.89
1:2	25.97 ± 2.44	48.06 ± 5.46	50.81 ±4.9
1:4	42.13 ± 10.19	77.73 ± 9.48	57.53 ± 4.53
1:6	60.85 ± 17.1	81.97 ± 2.97	71.79 ± 3.21
***Lc. casei* ATCC 393**			
Undiluted	30.04 ± 5.66	48.82 ± 5.19	48.66 ± 5.99
1:2	24.60 ± 7.11	47.93 ± 8.99	48.41 ±2.87
1:4	32.27 ± 4.78	63.5 ± 8.65	56.67 ± 3.61
1:6	68.57 ± 6.22	71.38 ± 9.19	60.14 ± 3.34
