# Supplementary figures and images for: Lacticaseibacillus casei Combats Biofilm Formation and Exhibits Antibacterial Activity Against Clinical Isolates of Staphylococcus aureus, Salmonella enterica, and Escherichia coli
Source: Microorganisms. 2025 Nov 24;13(12):2667. doi: 10.3390/microorganisms13122667 (PMC12735342; doi:10.3390/microorganisms13122667)

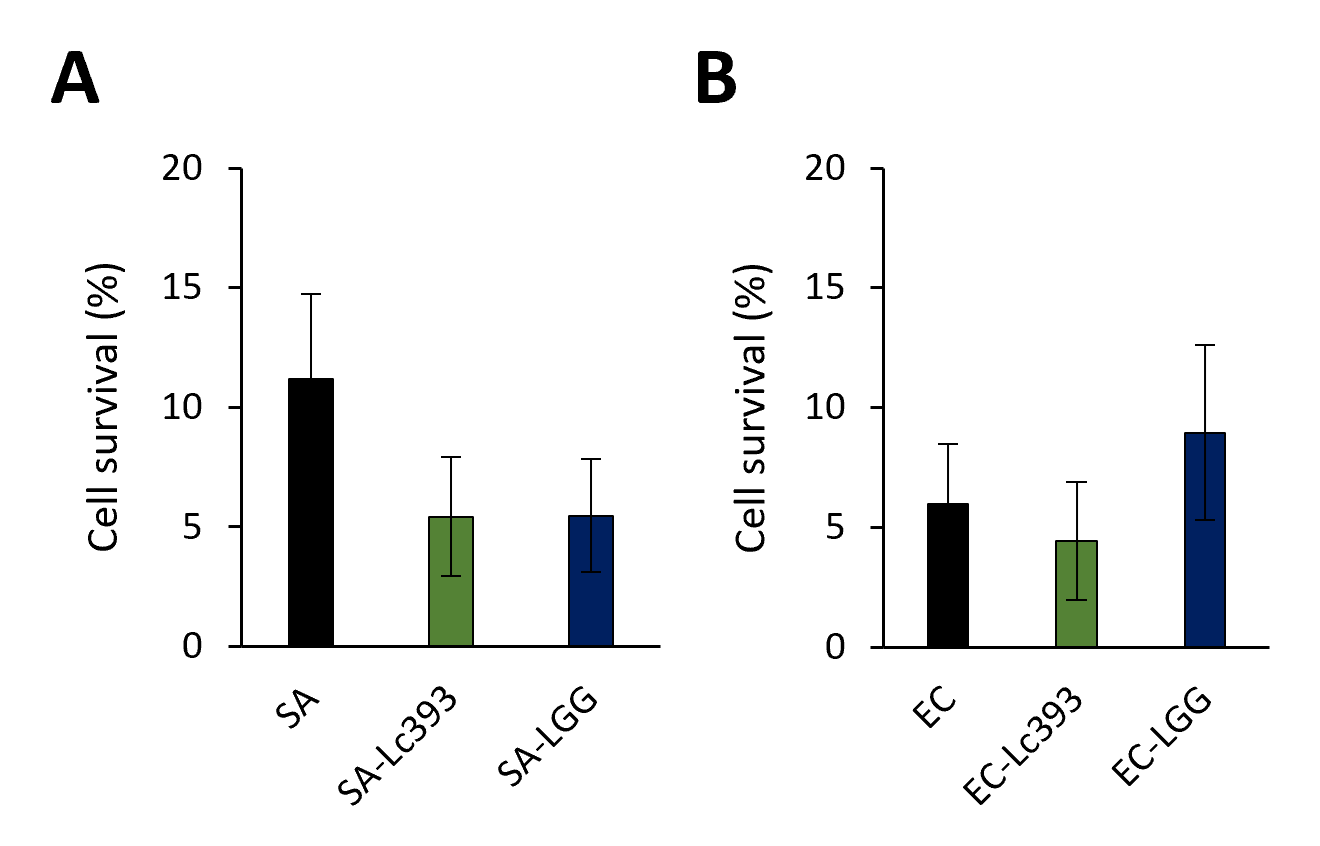

Supplement: Supplementary file 1 [file microorganisms-13-02667-s001.zip › Figure S1.tif]

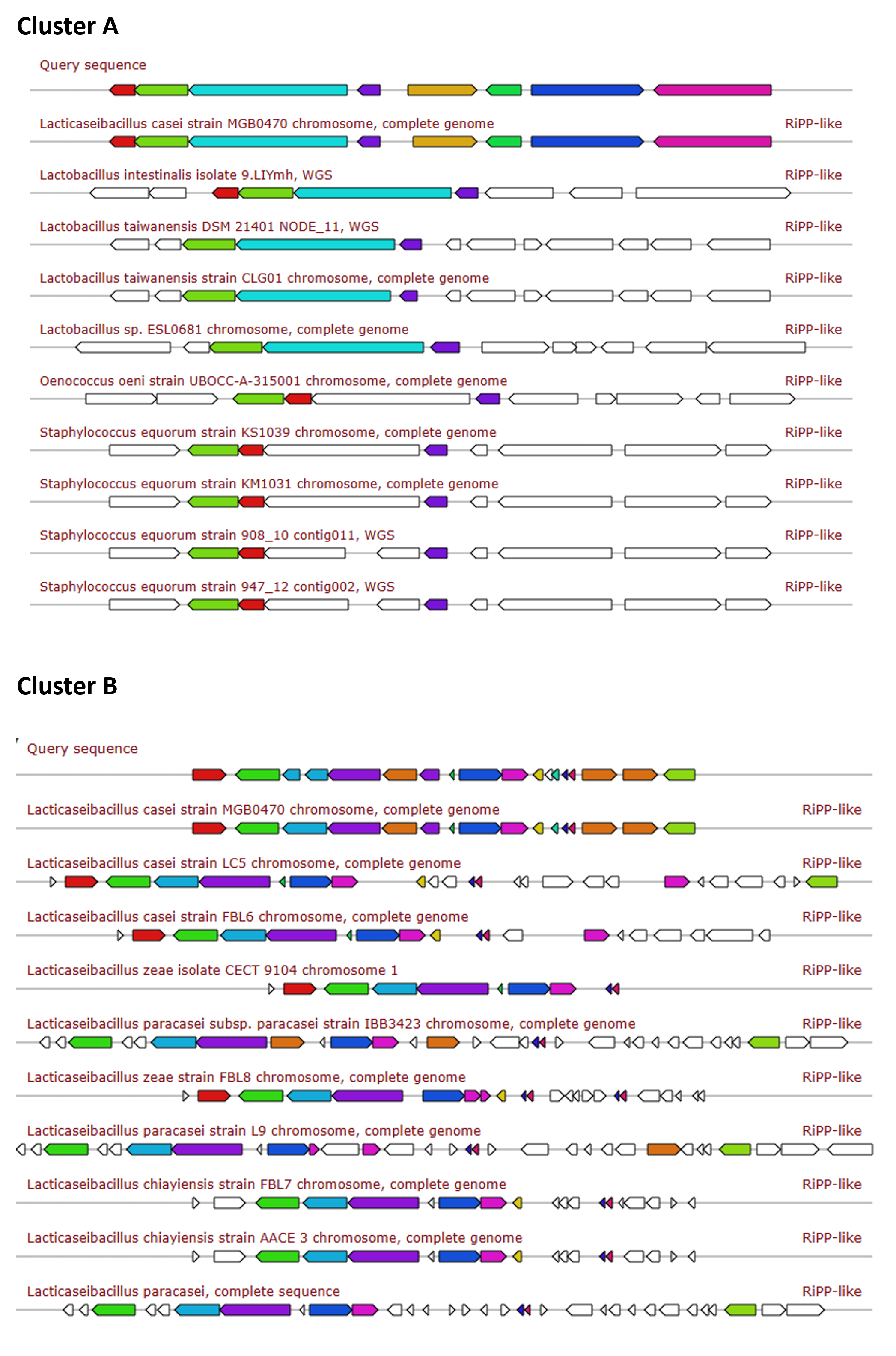

Supplement: Supplementary file 1 [file microorganisms-13-02667-s001.zip › Figure S2.tif]

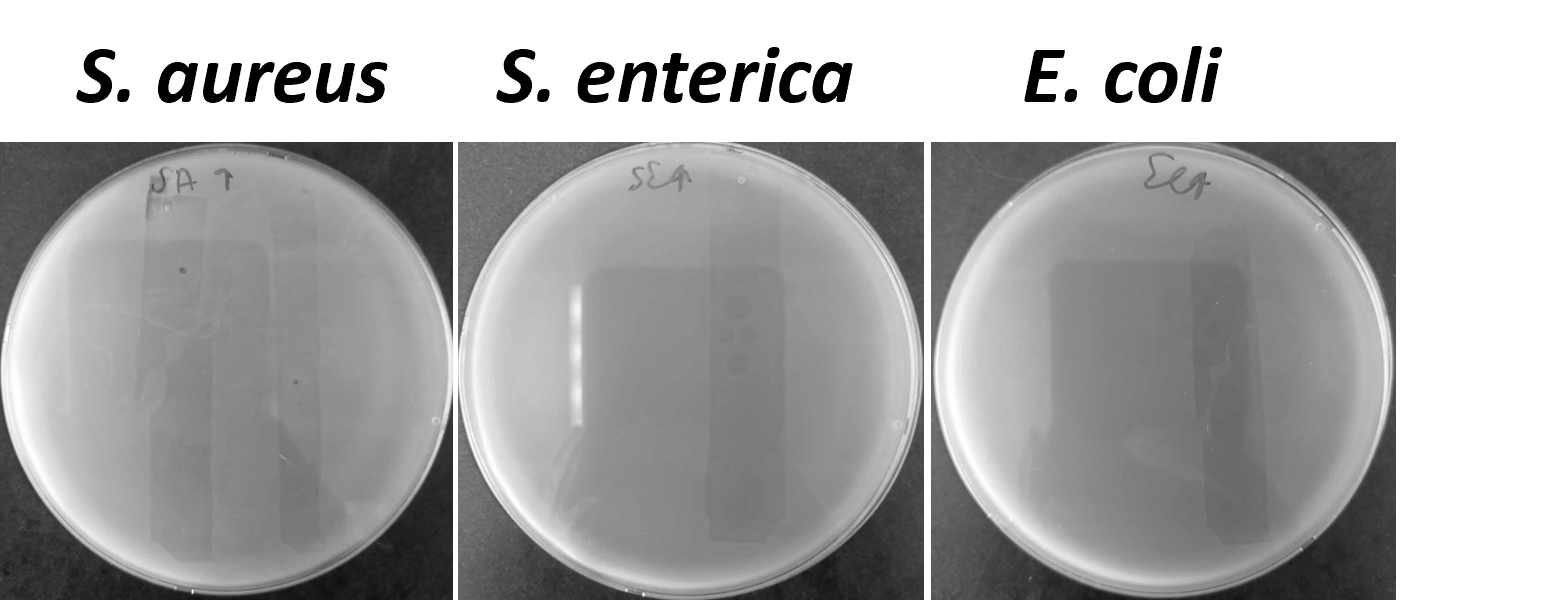

Supplement: Supplementary file 1 [file microorganisms-13-02667-s001.zip › Figure S3.tif]

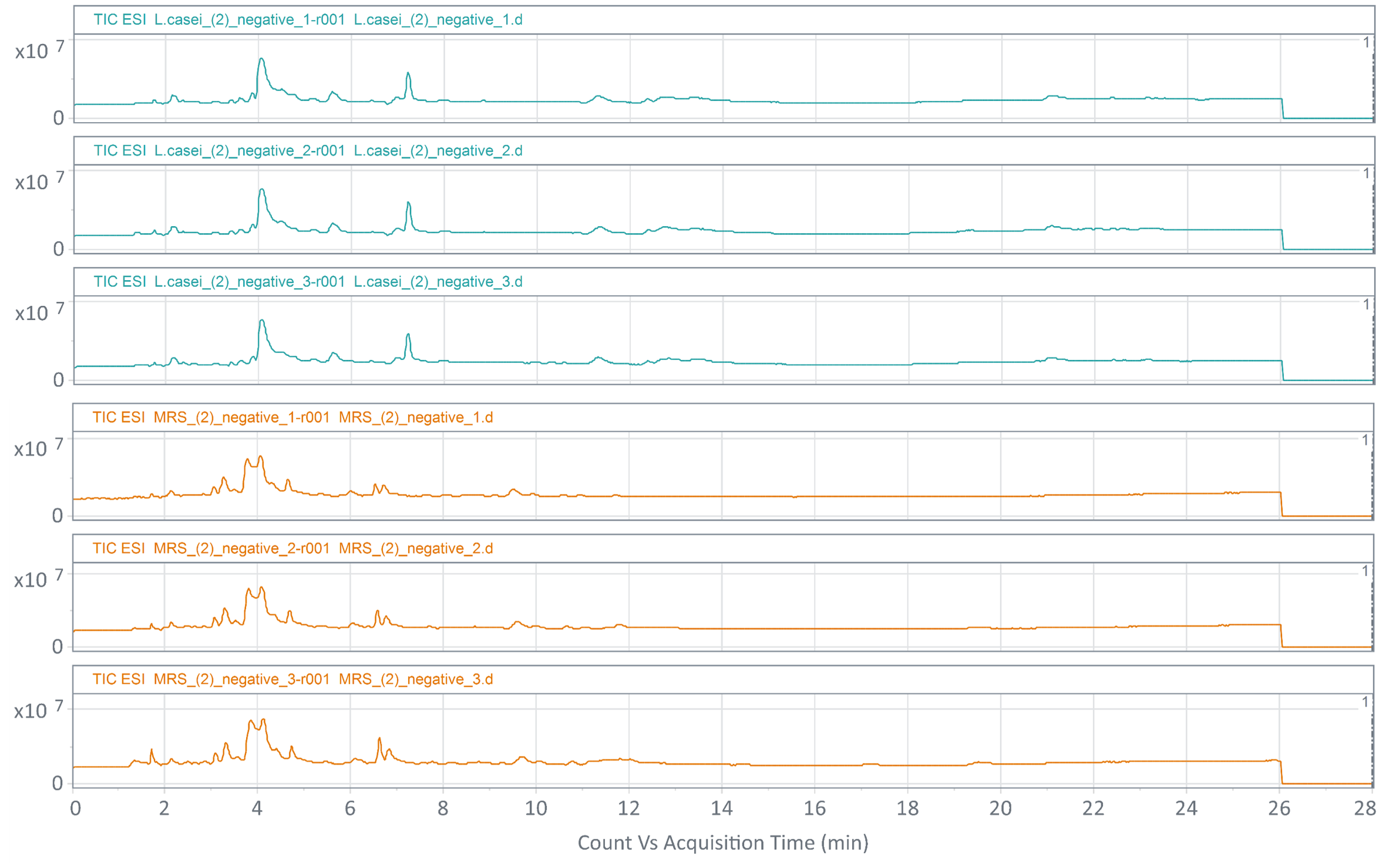

Supplement: Supplementary file 1 [file microorganisms-13-02667-s001.zip › Figure S4.tif]

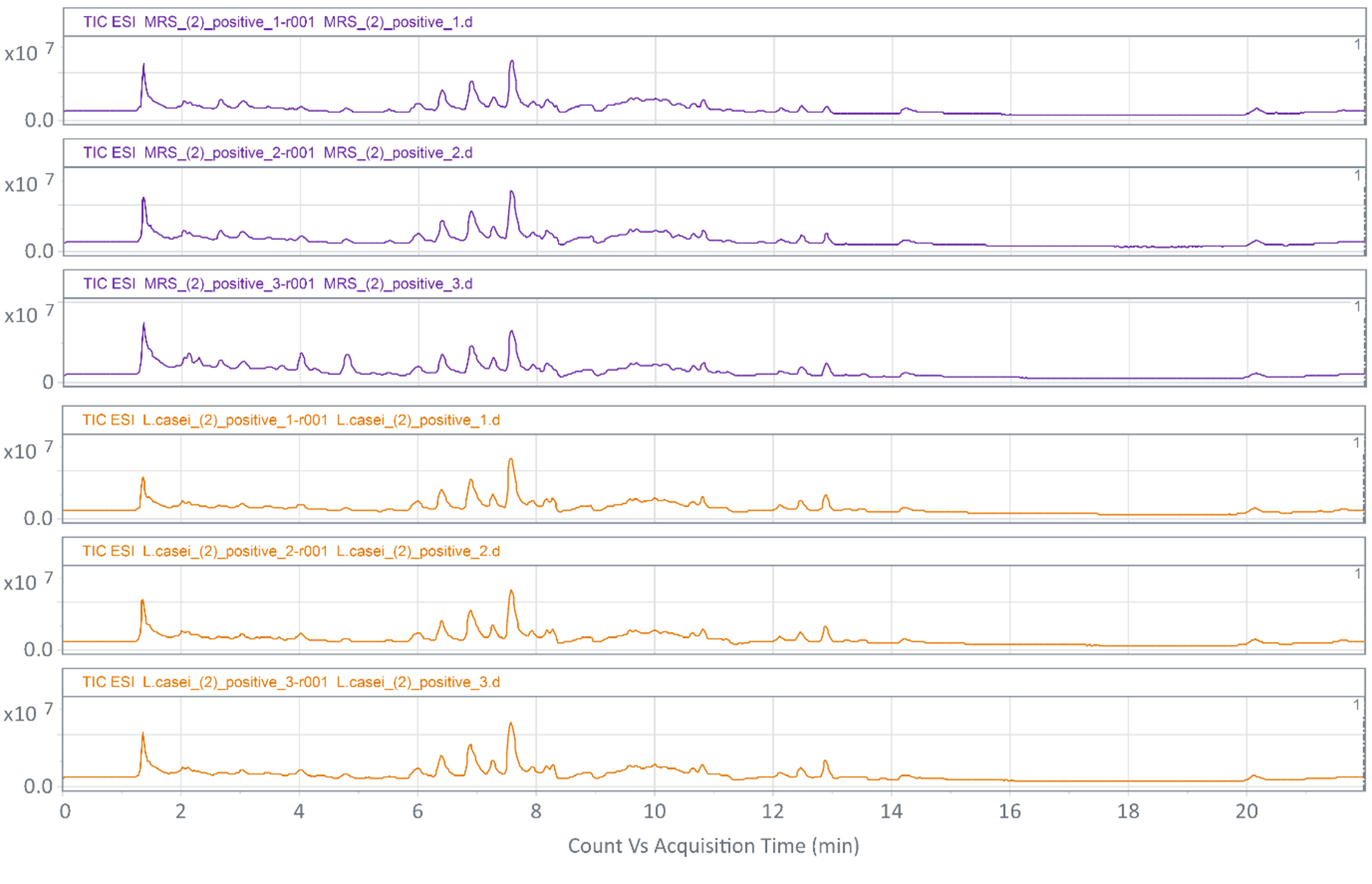

Supplement: Supplementary file 1 [file microorganisms-13-02667-s001.zip › Figure S5.tif]

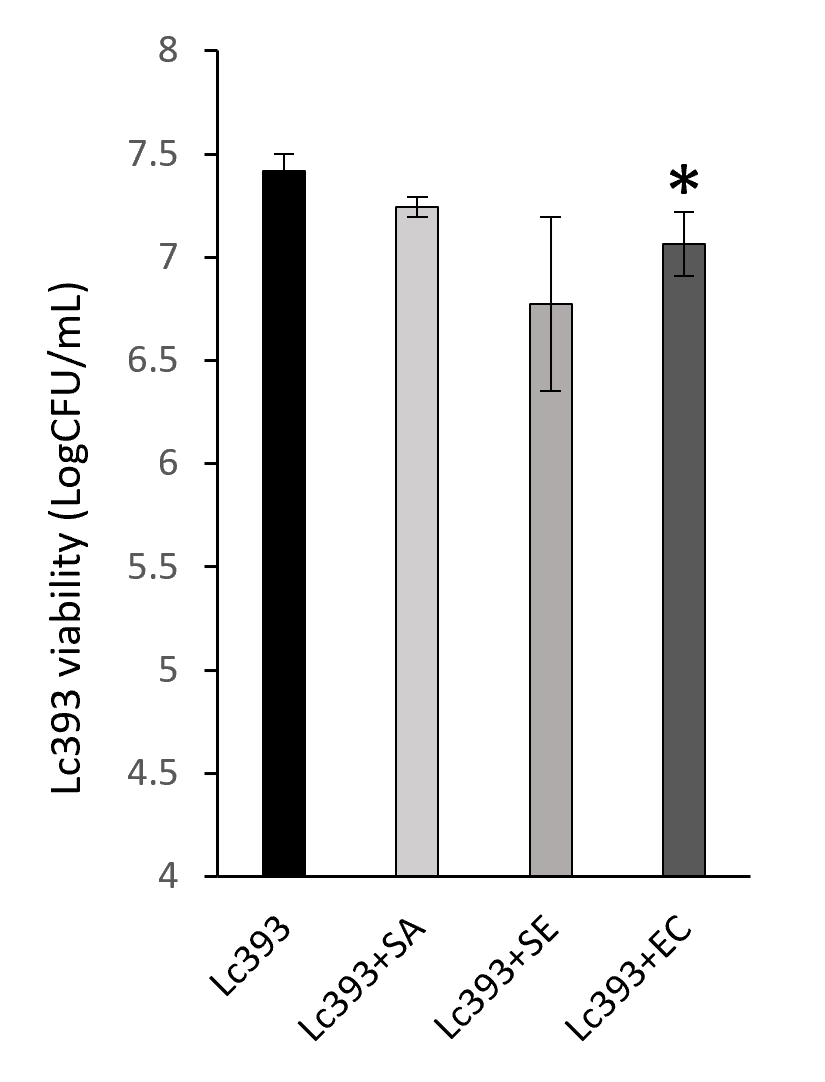

Supplement: Supplementary file 1 [file microorganisms-13-02667-s001.zip › Figure S6.tif]
